# Pancreatic cancer: challenges and opportunities

**DOI:** 10.1186/s12916-018-1215-3

**Published:** 2018-11-22

**Authors:** Huiyun Zhu, Tuo Li, Yiqi Du, Min Li

**Affiliations:** 1 Department of Gastroenterology, Changhai Hospital, Shanghai, 200433 China; 2 Department of Medicine, University of Oklahoma Health Sciences Center, Oklahoma City, OK 73104 USA; 3 Department of Endocrinology, Changzheng Hospital, Shanghai, 200003 China

**Keywords:** Pancreatic cancer, Diagnosis, Therapy

## Abstract

Pancreatic cancer is the fourth leading cause of cancer-related death in the United States, with increasing incidence. The mortality rate of pancreatic cancer is rising rapidly, and is projected to be the second most common of all malignant tumors by 2030. However, the diagnosis and therapy of pancreatic cancer remain a formidable challenge. Recently, enormous efforts have been made to develop several new methods for the early diagnosis and treatment of pancreatic cancer. We briefly introduce the most groundbreaking advances in pancreatic cancer diagnosis and clinical treatment strategies over the past 15 years, including surgery, chemotherapy, endoscopic therapy, immunotherapy and personalized medicine. The signaling pathways that are altered in the progression of pancreatic cancer, which may be used as therapeutic targets, are also discussed.

## Background

Despite the continuous decline in overall cancer death rates, the mortality rate of pancreatic cancer (PC) remains high. The latest cancer statistics show that PC is the third leading cause of cancer-related death in both males and females. Furthermore, the incidence of PC is on the rise, with approximately 44,330 deaths estimated in the United States in 2018 [1].

PC is a systemic disease with both locally advanced and metastatic phenotypes. It has a 5-year survival rate of approximately 8%. This disease is usually asymptomatic in the early stages, and without the use of invasive procedures, current screening methods are unable to achieve an effective early diagnosis. Only about 10% of PC patients are diagnosed at an early stage and are thus able to benefit from potentially curative surgical resection [1].

Recently, novel biomarkers, and new technologies and methods for early diagnosis, such as gastrointestinal endoscopy, have emerged. Surgical techniques and equipment have significantly improved, and new chemotherapeutic concepts and regimens have been tested. Preliminary immunotherapy studies have also been conducted in PC. Basic and translational research into its pathogenesis has further deepened our understanding of PC. On the 15^th^ anniversary of BMC Medicine, we provide a perspective on PC diagnosis and therapy in recent decades.

### Diagnosis: new biomarkers are still needed

Patients with PC most commonly present with abdominal pain, weight loss, asthenia, and anorexia, with some patients also having jaundice. Once a pancreatic mass is detected, contrast-enhanced computed tomography is sufficient to determine the initial stage and decide the treatment options. Contrast-enhanced endoscopic ultrasonography (CE-EUS), EUS elastography, or a biopsy of the mass accomplished by EUS-guided fine needle aspiration might help in the early detection of PC, especially for asymptomatic pancreatic masses that cannot be identified by imaging [2]. In addition, endoscopic retrograde cholangiopancreatography (ERCP) can provide tissue samples for diagnosis by guiding ductal brushing and lavage [2]. Currently available serum biomarkers, such as carcinoembryonic antigen and carbohydrate antigen 19-9, have been used in clinics for many years; however, their sensitivity is suboptimal. To date, there are no specific biomarkers for PC [2]. Novel and more sensitive biomarkers are still needed for early diagnosis of this deadly disease.

### Surgery: the cure

Surgery remains the only curative therapy for patients with PC. Even so, the impact of pancreatectomy on patient quality of life and long-term survival remains questionable. The surgical approach has evolved from what was, a few decades ago, a high-risk procedure, to a challenging, yet relatively safe, procedure today [3]. PC patients with stage I/II disease may benefit from surgical resection [4]. However, surgery alone is insufficient: without additional therapy, over 90% of patients relapse and die of their disease after surgery.

The poor outcomes associated with surgery alone mean that the roles of adjuvant therapies, such as chemotherapy and radiotherapy during or after the surgical procedure, have been extensively evaluated. For resectable tumors, surgery followed by adjuvant chemotherapy is the standard of care, with a 5-year survival rate of 30%. Patients with stage III borderline resectable cancers should undergo neoadjuvant therapy prior to resection.

### Chemotherapy and radiotherapy: a tough choice

For over a decade, gemcitabine was the standard first-line treatment for PC. However, in 2005, Moore et al. [5] reported that the addition of erlotinib could significantly improve overall survival and progression-free survival in advanced PC. Subsequently, the new chemotherapeutic regimens FOLFIRINOX, S-1, and nabpaclitaxel plus gemcitabine have shown improved survival benefit and are now the most commonly administered first-line therapies.

The role of adjuvant radiation therapy is controversial. Compared with chemotherapy alone, chemoradiation showed no survival benefit in patients with locally advanced PC [6]. Almost all patients will receive subsequent therapies with the inevitable disease progression that follows first-line therapy [7]. However, minimal data exist on second-line therapies for PC.

### Endoscopic therapy: minimal invasive

EUS and ERCP are the mainstays of interventional endoscopic practice. EUS offers a platform for a wide variety of direct tumor therapies, including the implantation of ^125^I seeds [8], celiac neurolysis [9], and fiducial placement for stereotactic body radiation therapy [10]. ERCP is an established endoscopic procedure that is important in elucidating indeterminate biliary structures and preoperative biliary drainage for patients with cancer of the pancreatic head [11]. Rational incorporation of endoscopic-based therapies into tumor studies may provide new hope for PC patients.

### Immunotherapy: role remains to be determined

Immunotherapy is considered to be a promising treatment for many cancer types [12]. The use of T cell checkpoints, including cytotoxic T lymphocyte protein 4 and programmed cell death protein 1 (PD-1), has shown much promise in several cancer types. However, PC is a notable exception [13]. In a phase I/II clinical trial [14], no positive effect was seen in PC patients who received a combination of the CXCR4 inhibitor ulocuplumab and anti-PD-1 therapy. The application of immunotherapy in PC is yet to be explored.

### Precision medicine and target therapy: opportunities and obstacles

Precision medicine is an emerging concept in oncology that offers improved outcomes by individualizing patient therapy. The complexity of the PC genome indicates that methods to individualize therapy are required. Sophisticated analyses using large numbers of tumors have revealed novel insights into PC pathophysiology [15]. The testing of novel precision medicine therapies by way of clinical trials is encouraged.

In the past few years, numerous targeted agents have emerged for PC treatment, including ECFR inhibitors (erlotinib) [5], VEGF and VEGFR inhibitors [16], phosphoinositide-3-kinase-mTOR pathway inhibitors, Janus kinase inhibitors, and Ras pathway inhibitors. However, most of these agents were ineffective, possibly because of the high molecular heterogeneity of the disease. An assessment of biomarkers is needed to identify the potential benefit from targeted therapy, and this may also provide important information to guide the use of precision medicine in clinical practice.

### Future novel perspectives

The tumor microenvironment has attracted much research interest in the past decade. The pancreatic tumor microenvironment contains an abundant fibrotic stroma, which includes a variety of cell types and extracellular matrix (ECM) components, such as collagen, fibronectin, hyaluronic acid, and N-acetyl-glucosamine. The stroma – not just a barrier for cancer cells – is critical in a variety of cellular processes, including tumor formation, invasion, metastasis, and drug resistance in PC. Recent studies have shown that PC stroma is associated with the modification of cancer cell metabolism, immune cell recruitment and the regulation of acinar-to-ductal metaplasia in the progression of PC. Further studies of ECM markers will guide the development of therapeutic targets for PC.

The most commonly mutated genes in PC are KRAS, TP53, SMAD4, and CDKN2A. However, none of these genes are currently druggable. Several new markers and therapeutic targets have been investigated, including mucins, mesothelin, and heavy metal transporters [17–19]. Recently, it has been shown that the zinc transporter ZIP4 is overexpressed in PC, and promotes tumor growth, metastasis, and cancer cachexia [20, 21]. Targeting ZIP4 might be a novel treatment strategy for PC patients with dysregulated zinc homeostasis.

## Conclusion

PC remains a challenging disease to treat. Although survival statistics for PC patients are currently bleak, our understanding of the complicated etiology and molecular mechanisms of PC has improved tremendously in recent years. Early identification of PC is the most desirable objective. Efforts should be made to determine appropriate biomarkers for early tumor detection, and to open new perspectives on immunotherapy. Precision medicine and multidisciplinary team collaboration should become a trend in the treatment of PC, and will deliver the best therapeutic schedule for individual patients.

## Abbreviations

ECM: Extracellular matrix
ERCP: Endoscopic retrograde cholangiopancreatography
EUS: Endoscopic ultrasonography
PC: Pancreatic cancer
PD-1: Programmed cell death protein 1
